# Telomere shortening induces aging-associated phenotypes in hiPSC-derived neurons and astrocytes

**DOI:** 10.1007/s10522-023-10076-5

**Published:** 2023-11-21

**Authors:** Jasmine Harley, Munirah Mohamad Santosa, Chong Yi Ng, Oleg V. Grinchuk, Jin-Hui Hor, Yajing Liang, Valerie Jingwen Lim, Wee Wei Tee, Derrick Sek Tong Ong, Shi-Yan Ng

**Affiliations:** 1 Institute of Molecular and Cell Biology, A*STAR Research Entities, Singapore, 138673 Singapore; 2 Department of Physiology, Yong Loo Lin School of Medicine, National University of Singapore, Singapore, 117593 Singapore

**Keywords:** Telomerase reverse transcriptase, Telomeres, Aging, hiPSC, Neurons, Astrocytes

## Abstract

Telomere shortening is a well-established hallmark of cellular aging. Telomerase reverse transcriptase (TERT) plays a crucial role in maintaining the length of telomeres, which are specialised protective caps at the end of chromosomes. The lack of in vitro aging models, particularly for the central nervous system (CNS), has impeded progress in understanding aging and age-associated neurodegenerative diseases. In this study, we aimed to explore the possibility of inducing aging-associated features in cell types of the CNS using hiPSC (human induced pluripotent stem cell) technology. To achieve this, we utilised CRISPR/Cas9 to generate hiPSCs with a loss of telomerase function and shortened telomeres. Through directed differentiation, we generated motor neurons and astrocytes to investigate whether telomere shortening could lead to age-associated phenotypes. Our findings revealed that shortened telomeres induced age-associated characteristics in both motor neurons and astrocytes including increased cellular senescence, heightened inflammation, and elevated DNA damage. We also observed cell-type specific age-related morphology changes. Additionally, our study highlighted the fundamental role of TERT and telomere shortening in neural progenitor cell (NPC) proliferation and neuronal differentiation. This study serves as a proof of concept that telomere shortening can effectively induce aging-associated phenotypes, thereby providing a valuable tool to investigate age-related decline and neurodegenerative diseases.

**Graphical abstract:**

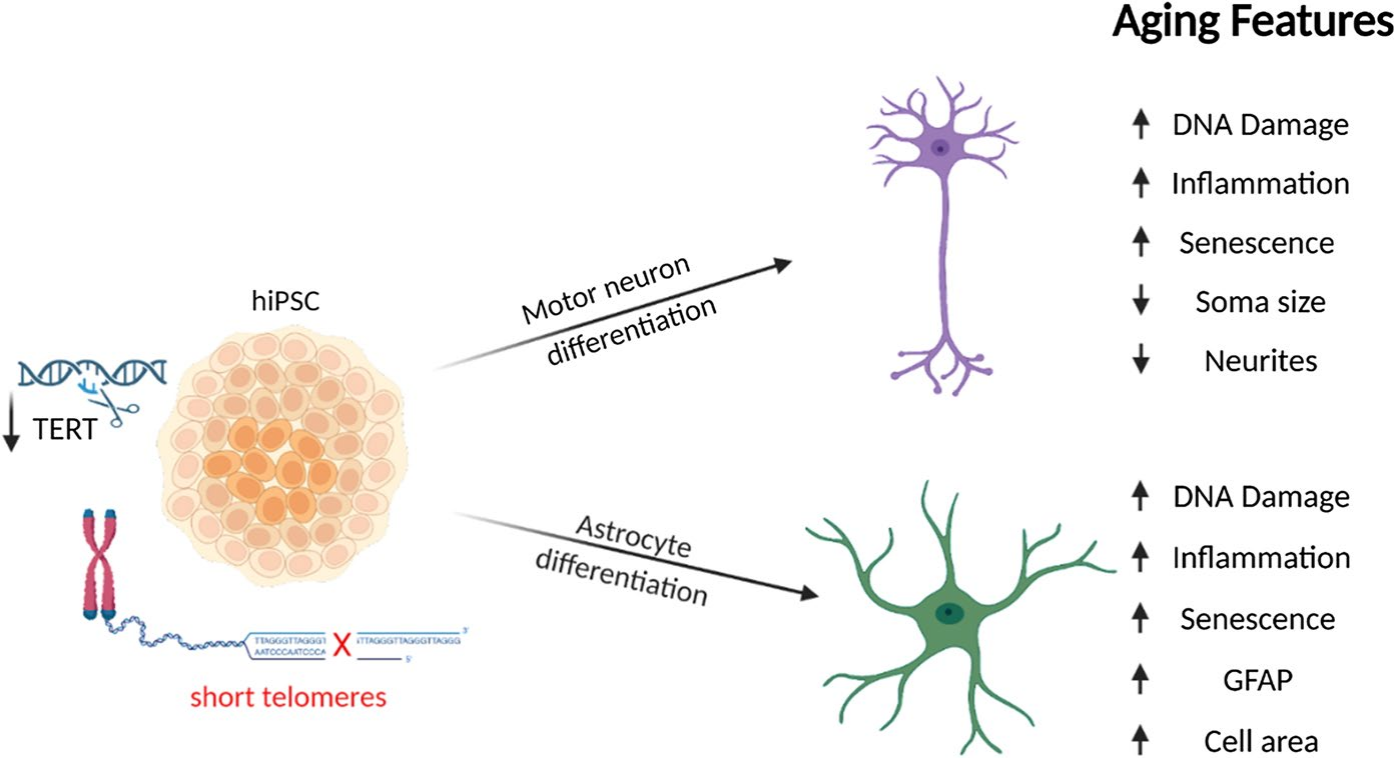

**Supplementary Information:**

The online version contains supplementary material available at 10.1007/s10522-023-10076-5.

## Introduction

Telomerase plays a crucial role in extending telomeres and mitigating telomere attrition, a key process associated with aging (Harley et al. 1990). Telomeres are specialised structures at the ends of chromosomes, serving to protect chromosome ends from DNA damage and degrading activities. Telomerase is a ribonucleoprotein (RNP) complex that elongates telomeres through the activity of reverse transcription. It consists of two main components; the catalytic subunit telomerase reverse transcriptase (TERT) and a telomerase RNA component (TERC) (Shay and Wright 2019; Nakamura et al. 1997; Feng et al. 1995). Through reverse transcription activity, telomerase elongates telomeres, preventing the accumulation of short telomeres that can lead to telomere dysfunction. Short telomeres activate DNA damage responses and other molecular mechanisms that contribute to cellular senescence or apoptosis. Consequently, short telomeres are considered one of the primary hallmarks of aging, as they lead to aging hallmarks including genomic instability, cellular senescence, and loss of regenerative capacity (López-Otín et al. 2023). Cell and animal models with genetic modifications or absence of telomerase activity have provided direct evidence for the role of telomerase in the maintenance of telomeres and its contribution to biological aging (Denham 2023). Moreover, telomeropathies, arising from mutations in genes responsible for telomere maintenance, manifest as premature aging syndromes (Vieri et al. 2021). Beyond telomere maintenance, TERT has been shown to have additional non-canonical functions in cellular processes such as proliferation, apoptosis, DNA repair, gene regulation, and modulation of oxidative stress responses (Thompson and Wong 2020; Ségal-Bendirdjian and Geli 2019).

Telomerase has an emerging role in the central nervous system. In the central nervous system (CNS), telomerase deficiency and telomere shortening have been associated with impaired neuronal differentiation, compromised neurogenesis, and increased vulnerability to age-related neurodegenerative diseases (Saretzki and Wan 2021; Liu et al. 2018; Levstek et al. 2020). Telomerase has shown to be present in both the developing and adult brain (Klapper et al. 2001; Caporaso et al. 2003). However, as neural precursor cells differentiate into neurons, telomerase activity rapidly decreases (Kruk et al. 1996; Cheng et al. 2007), and studies in mice have demonstrated that telomere shortening disrupts neuronal differentiation and neurogenesis (Ferrón et al. 2009). The presence of short telomeres has been linked to various neurodegenerative diseases (Rossiello et al. 2022), and mouse models with short telomeres have exhibited key features of neurodegeneration (Whittemore et al. 2019).

Human induced pluripotent stem cells (hiPSCs) have proven valuable for disease modelling and drug discovery, particularly for CNS disorders. However, the reprogramming of hiPSCs resets their cellular age and rejuvenates various age-related characteristics, posing challenges to model late-onset diseases like neurodegenerative disorders (Mahmoudi and Brunet 2012). Consequently, there is a need to develop strategies for inducing aging in hiPSC-based models to study CNS aging in both health and disease. Existing methods to artificially induce age-related changes in hiPSC are limited and include models that express progerin (Miller et al. 2013), pharmacological inhibition of telomerase (Vera et al. 2016) and the application of cellular stressors (Dong et al. 2017). However, there remains an unmet need to develop additional aging-induced models to further advance research in this field.

To generate an hiPSC based in vitro aging model, this study generated hiPSC lines with reduced telomerase activity and shortened telomeres. We address the role of telomere shortening in motor neurons and astrocytes by characterising the cellular and molecular effects. We demonstrate that telomere shortening has a role in both neurogenesis and aging and shortening telomeres can be used to induce aging-associated characteristics in different cell-types in vitro. Together this provides an important tool to investigate age-related decline in the CNS.

## Results

### Derivation and characterisation of hiPSCs with reduced telomerase activity and shortened telomeres

**Fig. 1:**
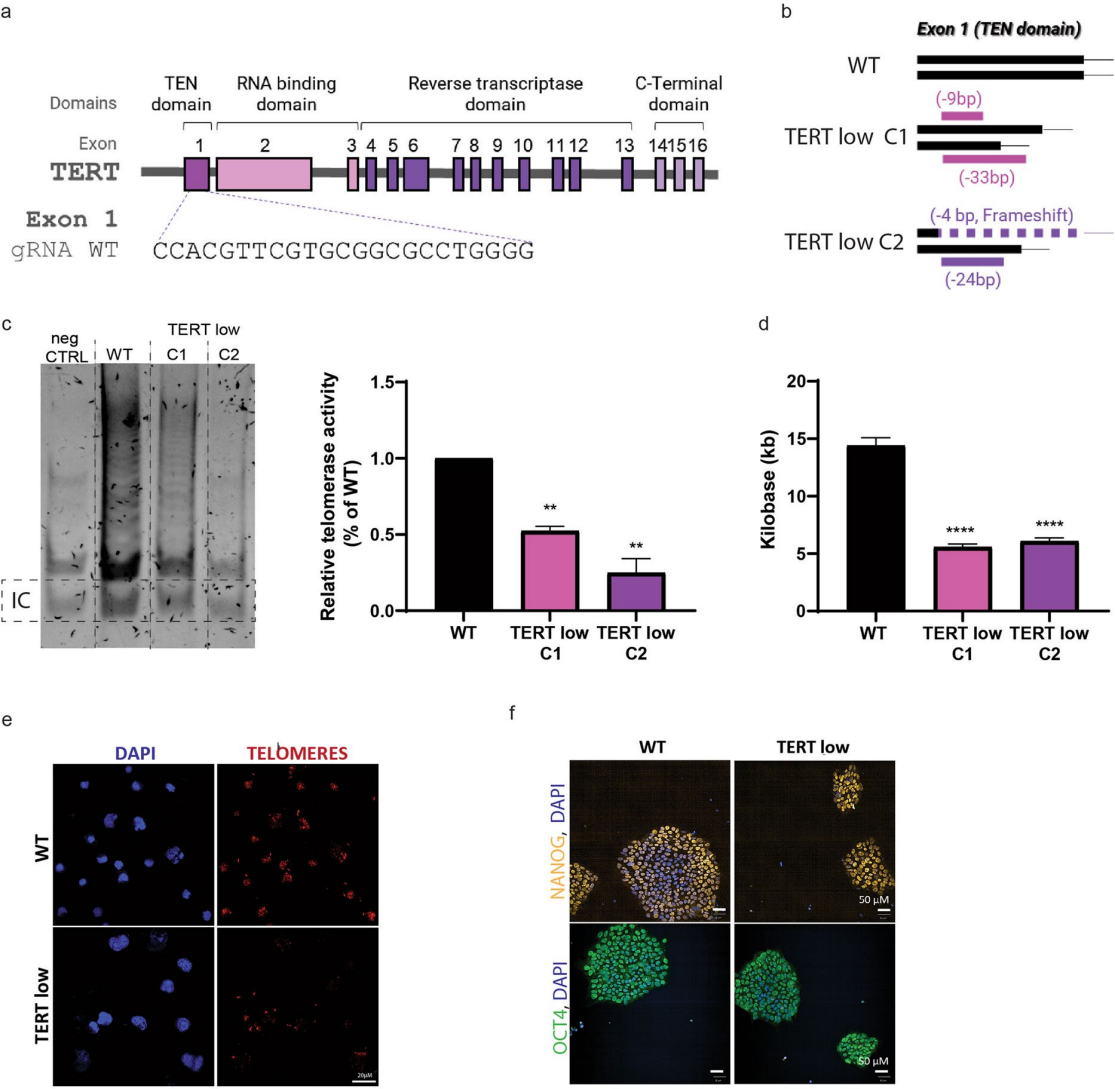
TERT deficient isogenic clone generation and characterisation. **a** Targeting strategy to generate TERT low hiPSC lines with guide RNA and CRISPR/Cas9. **b** Diagram of the two TERT low clones obtained, TERT low C1 and TERT low C2. **c** Telomerase activity of TERT low clones via TRAP assay visualisation and quantification show TERT low clones have reduced telomerase activity. Relative telomerase activity is calculated from the following formula [(TRAP sample—negative control)/Internal control (IC) of sample]/[(WT sample—negative control)/Internal control (IC) of WT sample)]*100. Data is shown as mean ± SD, n = 3, **P < 0.01; ANOVA with Tukey’s multiple comparisons test. **d** Average telomere length of WT and TERT low C1 and low C2 using the Absolute Human Telomere Length Quantification qPCR Assay Kit showing shorter telomeres in TERT low clones. Data is shown as mean ± SD, n = 3, ****P < 0.0001; ANOVA with Tukey’s multiple comparisons test. **e** Immunostaining of telomeres in WT and TERT low hiPSCs. TERT low clones show a decrease in telomeres compared to WT iPSCs. Scale bar = 20 μm. **f** Immunostaining of pluripotency markers NANOG and OCT4. Both WT and TERT low hiPSC lines display pluripotent markers. Scale bar = 50 μm

To investigate if reducing telomerase activity could induce an in vitro model of aging, we generated TERT deficient cell lines using CRISPR/Cas9 technology. Guide RNAs were targeted against exon one of the TERT gene and transfected into a wild type (WT) hiPSC line (Fig. 1a). Two TERT deficient clones were characterised, TERT low clone 1 (TERT low C1) has a 9 bp deletion and 33 bp deletion in each of the alleles of exon 1, and TERT low clone 2 (TERT low C2) has a 4 bp deletion and 24 bp deletion in each of the alleles of exon 1 (Fig. 1b).

To characterise if the TERT clones had a reduction in telomerase activity, the telomerase activity was checked by the Telomerase Repeated Amplification Protocol (TRAP) assay (Mender and Shay 2015). Both TERT low C1 and TERT low C2 displayed reduced telomerase activity (Fig. 1c, Supp Fig. 1a). TERT low C1 exhibited residual activity whereas C2 showed an almost complete loss of activity (Fig. 1c). The residual activity in C1 could be accountable to the 9 bp deletion in one allele, leading to an inframe deletion of 3 amino acids, which may not be sufficient to completely abolish TERT function. On the other hand C2 has a 4 bp frameshift mutation and 24 bp deletion in each of the alleles, which are more likely to disrupt TERT function. To explore if the reduced telomerase activity was accompanied with shortened telomeres, the average telomere lengths were assessed using the Absolute Human Telomere Length Quantification qPCR Assay Kit. The average telomere length was significantly shorter for the TERT low clones, with TERT low C1 displaying a 61% decrease and TERT low C2 a 58% decrease in telomere length (TERT low C1, 5.6 ± 0.26 kilobase (kb), TERT low C2, 6.08 ± 0.29 kb) compared to WT (14.4 ± 0.69 kb) (Fig. 1d). This was maintained across multiple cell passages (Supp Fig. 1b). Interestingly, TERT low C1 exhibited residual activity in the TRAP assay, yet its telomere length decreased. This suggests that the mutation might not completely disrupt all active sites or substrate binding regions. However, it’s important to acknowledge that these mutations, despite preserving some activity, may still impair the overall enzyme’s function given the complexity of telomerase’s role in maintaining telomeres and cellular function. Visualisation of telomeres with immunofluorescence staining further confirms a decrease in telomeres in the TERT low hiPSC clones (Fig. 1e).

To ensure TERT low hiPSCs maintain pluripotency, pluripotency markers OCT4 and NANOG were examined by immunocytochemistry. TERT low C1 and C2 hiPSC formed colonies as expected (Supp Fig. 1c) and were both positive for both OCT4 and NANOG (Fig. 1f), indicating a pluripotency state. TERT low clones were observed to have decreased proliferation, supporting the observed absence of telomerase activity (Supp Fig. 1d).

### Motor neurons with shortened telomeres show dysregulated neurogenesis

**Fig. 2:**
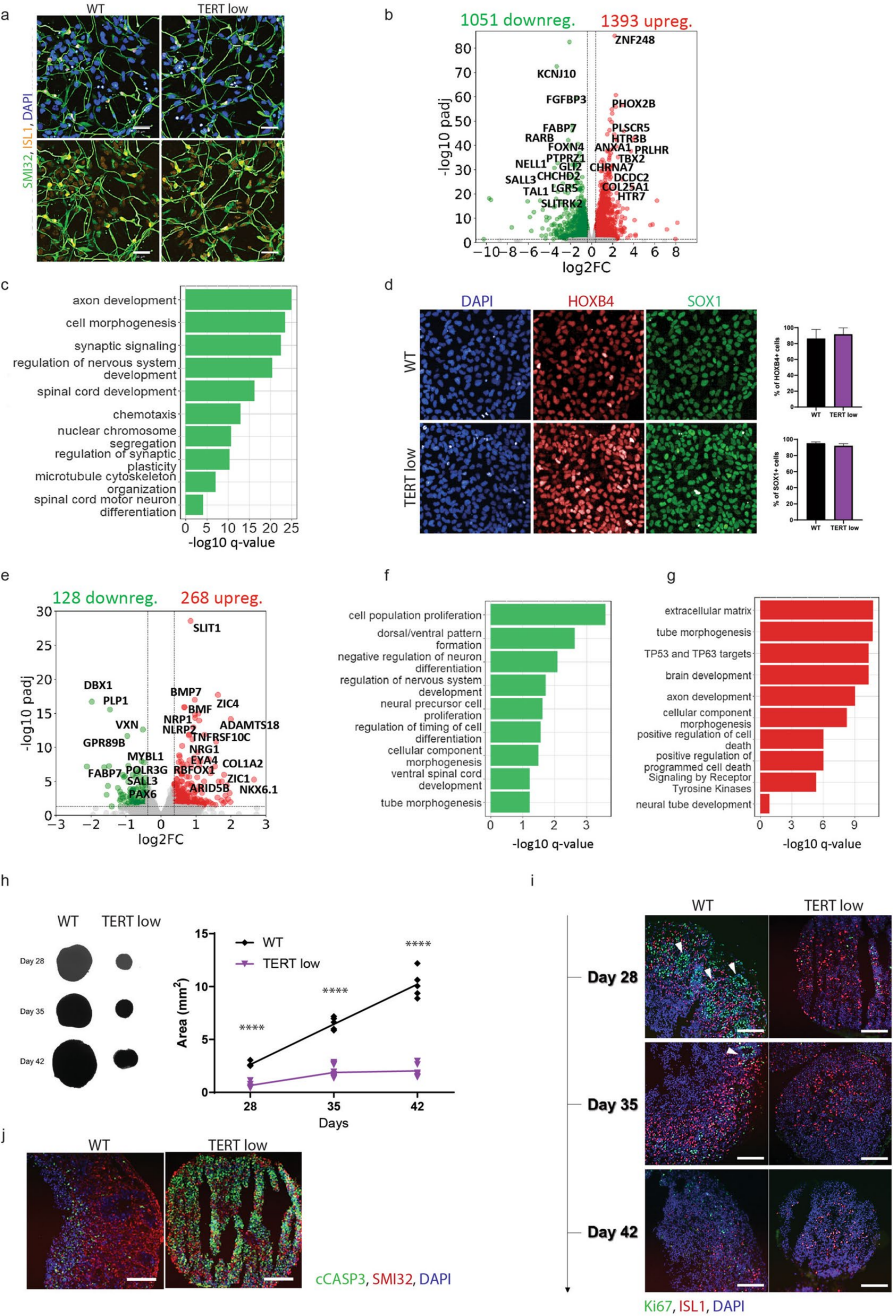
Telomere shortening disrupts motor neurogenesis. **a** WT and TERT low day 28 motor neurons, immunostained with DAPI and motor neuron markers ISL1 and SMI-32. Scale bar = 50 μm. **b** Differentially expressed genes (DEG)s in TERT low motor neurons compared to WT motor neurons. **c** Downregulated pathways in TERT low motor neurons. **d** Immunostaining of HOXB4 and SOX1 in NPCs. NPCs are positive for neural progenitor markers. Scale bar = 50 μm. Quantification of HOXB4 and SOX1 positive cells show no difference in differentiation to NPC fate between WT and TERT low NPCs. Data shown is mean ± SD, n = 3, P = ns; unpaired T-Test. **e** Differentially expressed genes (DEG)s in TERT low NPCs compared to WT NPCs. **f** Down regulated pathways TERT low NPCs. **g** Up regulated pathways in TERT low NCPs. **h** Spinal organoid size and measurement showed TERT low organoids are reduced in size. Data is shown as individual measurements for each organoid (n = 6 minimum), with the mean displayed by the line, ****P < 0.0001; ANOVA with Tukey’s multiple comparisons test. **i** Representative immunostained images of Ki67, ISL1 and DAPI in spinal cord organoids show reduced proliferation and ISL1 + motor neurons in TERT low organoids. White arrows show neural rosette structures. Scale bar = 200 μm. **j** Representative immunostained images of cleaved caspase 3 (cCASP3), SMI-32 and DAPI show increased apoptosis in TERT low organoids. Scale bar = 200 μm

To investigate the impact of reduced telomerase activity and telomere shortening in motor neurons, we generated spinal cord motor neurons from WT and TERT low C2 hiPSCs using a previously established protocol (Supp Fig. 2a) (Hor et al. 2021). These motor neurons were positive for motor neuron specific markers ISLET1 (ISL1) and SMI-32 (Fig. 2a) Subsequently, we performed RNA sequencing on poly(A) + selected mRNA libraries isolated from TERT low and WT motor neurons to gain insights into the disrupted biological pathways. Quantification of TERT expression showed TERT to be very lowly expressed in WT motor neurons (WT; Transcripts per million (TPM) = 0.15 ± 0.16) with a decrease of expression in TERT low motor neurons (TERT low; TPM = 0.017 ± 0.02). TERT low motor neurons with shortened telomeres exhibited extensive dysregulation of gene expression with 1051 down-regulated and 1393 up-regulated genes (Fig. 2b). Down-regulated pathways were predominantly associated with neurogenesis, neuron and nervous system development, neuron projection and axon development, cell morphogenesis and synaptic signalling (Fig. 2c). These findings align with previous research demonstrating that telomere shortening disrupts neuronal differentiation and neurogenesis in the mouse brain (Ferrón et al. 2009), providing further evidence of the essential role of telomeres in neuronal development.

For a deeper understanding of the role of shortened telomeres in motor neurogenesis we generated day 10 neural progenitor cells (NPCs) from WT, TERT low C1 and TERT low C2 hiPSCs. This cell population was positive for SOX1 + and HOXB4 + cells, indicating an NPC population with a spinal cervical identity (Fig. 2d). We performed RNA sequencing on poly(A) + selected mRNA libraries isolated from TERT low and WT NPCs (day 10). TERT expression is approximately eightfold greater in NPCs (WT NPC; TPM = 1.18 ± 0.02) compared to motor neurons (WT; TPM = 0.15 ± 0.16). As expected, TERT was shown to be significantly down regulated in both TERT low clones at the NPC stage (C1; fold change = −2.9, padj =  < 0.0001, C2; fold change = −1.68, padj = 0.02, combined; fold change = −1.10, padj = 0.0027). Differential gene analysis showed 128 down and 268 up- regulated differential expressed genes (DEGs) in TERT low NPCs (Fig. 2e). S pinal cord development and neural tube patterning pathways were observed to be down regulated (Fig. 2f). Alongside a downregulation of cell proliferation pathways, including genes PAX6 and FOXG1, which play crucial roles in regulating neural proliferation and differentiation (Quintana-Urzainqui et al. 2018). We also observed a decrease in Ki67, a proliferation marker, in TERT low NPCs through immunocytochemistry (Supp Fig. 2b). This suggests that TERT and shortened telomeres may influence neural cell proliferation by regulating PAX6 expression, consistent with previous findings (Kim et al. 2017).

TERT low NPCs exhibited a notable upregulation of pathways related to development and neuronal differentiation, including tube and embryonic morphogenesis, axon development, and responses to growth factors (Fig. 2g). Notably, we observed an upregulation of 45 genes transcriptionally activated by p53 and 30 genes activated by homolog p63, both of which have been associated with TERT, telomerase activity and downstream aging pathways (Vorovich and Ratovitski 2008; Jin et al. 2010). Additionally, several pathways involved in extracellular matrix (ECM) organisation, cellular adhesion, and cell death and apoptosis were also upregulated (Fig. 2g). The increase in cell death pathways was further confirmed by assessing cell death at different time points. Although an increased cell death was not observed at D10, TERT low NPCs displayed a significant increase in cell death by D17 which was examined by Annexin V apoptosis assay (Supp Fig. 2c). The most highly upregulated gene was NKX6.1, a homeobox gene known to increase during motor neuron development and play a critical role in neuronal fate determination in the spinal cord (Sander et al. 2000; Li et al. 2016). This finding underscores the potential involvement of TERT and shortened telomeres in regulating essential genes that govern neuronal development and fate specification.

To phenotypically validate the role of short telomeres in motor neurogenesis, we generated spinal cord organoids to examine structural and neural development changes, resembling those in human 3D structures. The spinal organoids were generated by encapsulating day 10 WT NPCs and TERT low C2 NPCs in Matrigel, which were then matured in a spinner flask until day 28, 35, and 42 (Supp Fig. 2d). TERT low organoids consistently exhibited a smaller size compared to control organoids at all examined time points (Fig. 2h). To further elucidate the underlying mechanisms, we performed sectioning and immunolabeling on these organoids. Our analysis revealed a decrease in the number of proliferating cells, evidenced by a reduction in Ki67 + cells (Fig. 2i). Concurrently, we observed an increase in cell death, indicated by elevated expression of cleaved caspase 3 (cCASP3) (Fig. 2j), thus corroborating the findings from the RNA sequencing analysis conducted at the NPC stage. Furthermore, neural rosette structures, which are the developmental signature of neural progenitors in cultures, were no longer observed in TERT low spinal organoids (Fig. 2i). This observation further reinforces an essential role for telomeres in motor neurogenesis and spinal cord development.

### Motor neurons with shortened telomeres show age-dependent phenotypes

**Fig. 3:**
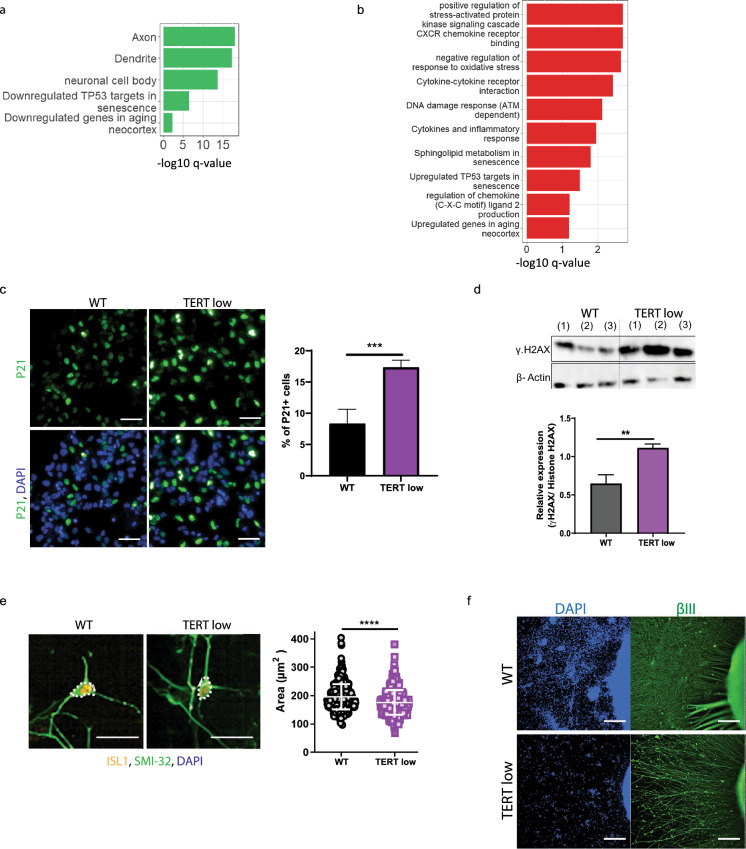
Age-dependent characteristics are observed in motor neurons with shortened telomeres. **a** Down regulated aging related pathways in TERT low motor neurons. **b** Up regulated aging related pathways in TERT low motor neurons. **c** Representative immunostaining images of increased P21 in TERT low motor neurons and quantification. Scale bar = 50 μm. Data is shown as mean ± SD, n = 3, ***P < 0.001; unpaired T-Test. **d** Western blot images indicating increased γ.H2AX in TERT low motor neurons with its relative quantification over total H2AX. Data is shown as mean ± SD, n = 3, ***P < 0.001; unpaired T-Test. **e** Representative images of soma size analysis and quantification displays a decrease in soma size in TERT low motor neurons. Data is shown as cell by cell measurements, with mean ± SD, n = 3, ****P < 0.0001; unpaired T-Test. **f** WT and TERT low spinal cord organoids immunolabeled with βIII-tubulin and DAPI, show TERT low organoids have reduced density and complexity of neurite outgrowth. Scale bar = 500 μm

Short telomeres are considered one of the primary hallmarks of aging, and have shown to lead to additional aging characteristics. Therefore, we further analysed the RNA sequencing data from TERT low and WT motor neurons to investigate if pathways closely linked with aging were present. ays asIndeed, motor neurons with shortened telomeres displayed an alteration of pathwsociated with aging phenotypes, including, cellular senescence, inflammation pathways (chemokine and cytokine release), DNA damage pathways, and cellular stress (Fig. 3a, b). There was also a downregulation of genes associated with neuronal morphology (Fig. 3a), and neuronal cell shrinking and axon and dendritic shrinking has been associated with aging (Blinkouskaya et al. 2021). Furthermore, analysis of the RNA sequencing data, revealed a notable correlation between dysregulated genes in TERT motor neurons and genes that undergo expression changes in the neocortex of aged adult mice (Fig. 3a, b). Supporting this, many age associated genes were dysregulated (e.g., TP53, LMNA, FOXO1) with 42 of the DEGs found in the GeneAge gene list (de Magalhães and Toussaint 2004).

To complement the gene expression changes, we wanted to investigate if age-related characteristics could be observed in motor neurons with shortened telomeres. As cellular senescence is a key characteristic of aging and there was a notable alteration of p53 signalling leading to senescence (up; A2M, CCND2, CDKN1A, SNCA, ABCA8, ADAMTS5, down; BUB1, CDC20, NEK2, TOP2A, TTK, CCNB2, AURKB, KIF20A, SPAG5, UBE2C, TPX2, KIF4A), the presence of cyclin-dependent kinase (CDK) inhibitor p21 (P21), a widely used marker of cellular senescence was examined by immunocytochemistry in the motor neurons. TERT low motor neurons showed an increase in P21-positive cells, indicating that short telomeres increase cellular senescence (Fig. 3c). As DNA damage accumulation is a hallmark of aged cells and there was an upregulation of ataxia-telangiectasia mutated (ATM) DNA damage response genes in motor neurons with shortened telomeres, we examined double-strand DNA breaks. Quantification of phosphorylated H2AX, an ATM-dependent early cellular response to DNA double-strand breaks (Kobayashi et al. 2009; Zha et al. 2008), revealed that telomere shortening led to a significant upregulation of γ.H2AX, indicating an increase in DNA damage and supporting the RNA analysis (Fig. 3d).

Neuronal soma size has been indicated to predict neuronal health (Dukkipati et al. 2018) and a smaller soma size has been associated with aged and diseased tissues (Liu et al. 1996; Castro et al. 2023). In the TERT low motor neurons there was a significant downregulation of 50 genes that have been associated with the neuronal cell body (Supp Fig. 3a). Therefore, we visualised neuronal morphology by SMI-32 immunocytochemistry and measured the size of the neuronal cell body (Fig. 3e). Motor neurons with short telomeres displayed a decrease in soma size (Fig. 3e). In addition, neurite degeneration is an age-associated characteristic (Salvadores et al. 2017), and we observed a down regulation of genes associated with axons and dendrites. Interestingly, we observe a down regulation of Forkhead Box O 1 (FOXO1) (fc = 0.50, padj = 0.0039), a transcription factor that has shown to inhibit age-progressive axonal degeneration (Hwang et al. 2018). To investigate this in vitro, we examined neurite outgrowth density from spinal cord organoids. TERT low spinal cord organoids displayed a decrease in neurite outgrowth from the organoid, with a reduction in both neurite density and length (Fig. 3f). Together these data support shortened telomeres in motor neurons induce age-dependent characteristics.

### Astrocytes with shortened telomeres show cell-type specific aging characteristics

**Fig. 4:**
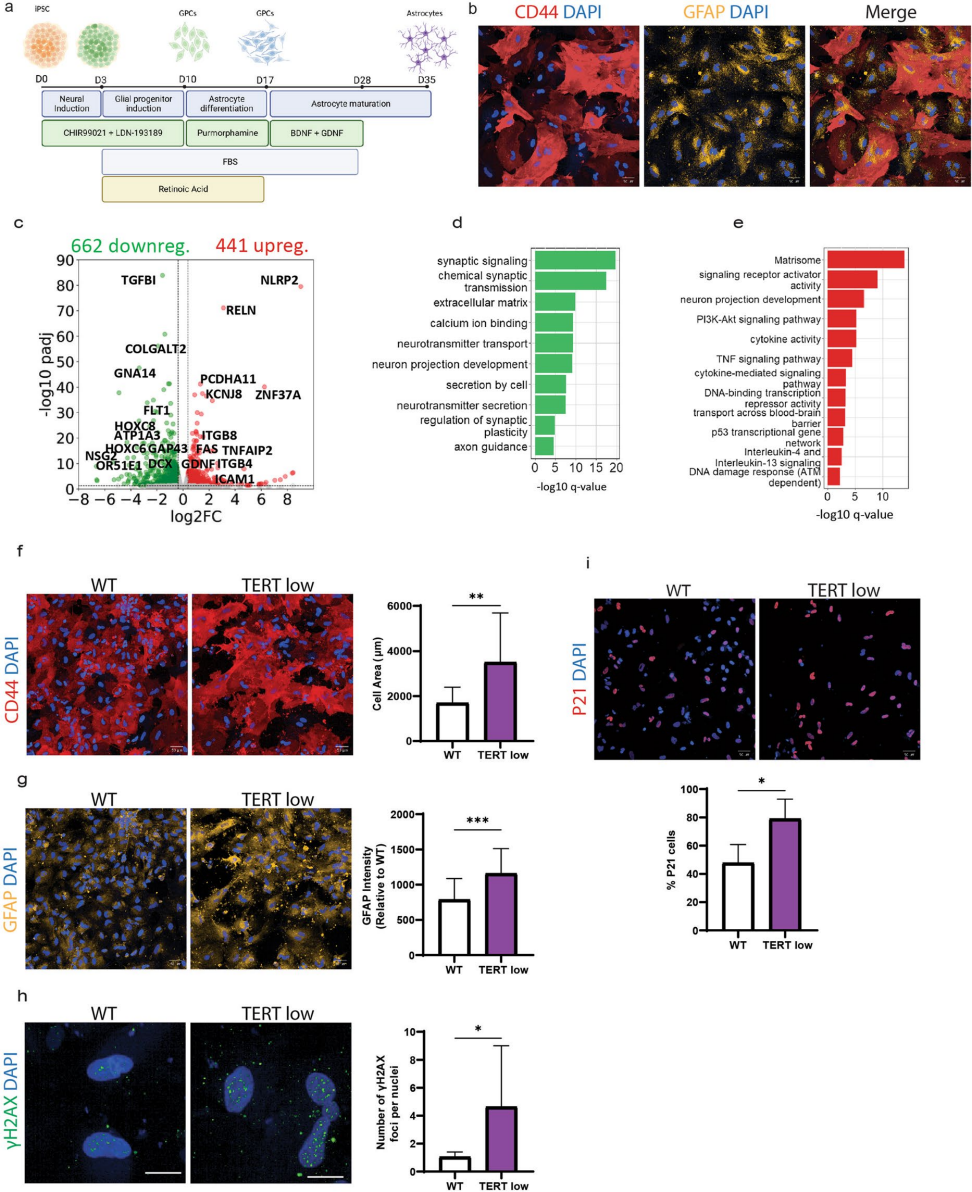
Astrocytes with short telomeres display aging characteristics. **a** Astrocyte differentiation protocol. Schematic of astrocyte differentiation from hiPSCs. **b** WT astrocytes, immunostained with astrocyte markers CD44 and GFAP and DAPI. **c** Differentially expressed genes (DEGs) in TERT low astrocytes. **d** Down regulated pathways in TERT low astrocytes. **e** Up regulated pathways in TERT low astrocytes. **f** WT and TERT low astrocytes immunolabeled with CD44 with quantification of cell size show TERT low astrocytes have an increase in cell size. Scale bar = 50 μm. Data is shown as mean ± SD, n = 6, **P < 0.01; unpaired T-Test. **g** WT and TERT low astrocytes immunolabeled with GFAP. Quantification of GFAP intensity shows TERT low astrocytes have an increase in GFAP. Scale bar = 50 μm. Data is shown as mean ± SD, n = 6, ***P < 0.001; unpaired T-Test. **h** Representative immunostaining images of γ.H2AX and the quantification of the number of γ.H2AX foci show an increase of γ.H2AX foci in TERT low astrocytes. Data is shown as mean ± SD, n = 4, *P < 0.05; unpaired T-Test. Immunolabeling of P21 expression in WT and TERT low astrocytes and the percentage of P21 cells show TERT low astrocytes have an increase in P21 expression. Scale bar = 50 μm. Data is shown as mean ± SD, n = 4, *P < 0.05; unpaired T-Test

As more age-induced changes have been observed in non-neuronal cells (Allen et al. 2023), and to explore if the aging effects of telomere shortening were motor neuron-specific, we generated human spinal cord astrocytes (Fig. 4a). We were able to generate highly enriched populations of hiPSC-derived astrocytes from WT, TERT low C1 and TERT low C2 hiPSCs which expressed the astrocytic markers glial fibrillary acidic protein (GFAP) and CD44 in 35 days (Fig. 4b). It has previously been reported that TERT protein is not present in GFAP-positive astrocytes (Spilsbury et al. 2015), and supporting this we could not detect TERT expression in the wild-type astrocytes. Therefore, we hypothesised if aging phenotypes were observed in the astrocytes it could be due to shortened telomeres.

To investigate cellular and molecular changes we performed RNA sequencing on poly(A) + selected mRNA libraries isolated from TERT low astrocytes and the WT control line. Differential gene expression analysis of TERT low and WT astrocytes revealed 662 down-regulated genes and 441 up-regulated genes in TERT low astrocytes (adjusted P-value < 0.05) (Fig. 4c). Notably, the down-regulated pathways in TERT low astrocytes included those involved in the regulation of synapse formation and function, particularly neurotransmitter transport and secretion (Fig. 4d). These findings align with previous research showing altered expression of synapse-regulated genes and reduced synaptic transmission in aged mouse brains astrocytes (Boisvert et al. 2018; Kawano et al. 2012), and down-regulation of synaptic transmission genes in the aged human brain (Wruck and Adjaye 2020). Moreover, we observed a significant downregulation of genes associated with synaptic plasticity, a phenomenon that has shown to decline with age in astrocytes (Popov et al. 2021). Additionally, our study identified a significant downregulation of genes involved in calcium regulation pathways. Previous research has highlighted deceased astrocytic Ca^2+^ transients with aging (Gómez-Gonzalo et al. 2017; Lalo et al. 2018; Ding et al. 2022), and our findings further support these observations. Interestingly, cell division cycle protein 20 (CDC20) was shown to be down regulated in TERT low astrocytes (fc = −1.33, padj = 0.039) and TERT low motor neurons (fc = −0.45, padj = 0.0035) and recent studies have shown CDC20 downregulation to cause premature aging and cellular senescence (Fujita et al. 2020; Volonte et al. 2022).

An upregulated pathway of interest was the matrisome, which encompasses genes encoding ECM and ECM-related proteins, including collagens, glycoproteins, integrins and proteinases (Fig. 4e). The upregulation of the ECM genes and ECM organisation has also been reported in the aged human brain (Wruck and Adjaye 2020). Astrocytes have an essential function in regulation of the blood brain barrier (BBB) and the progressive declines in BBB have been associated with advancing age (Preininger and Kaufer 2022). Here we observe an upregulation of genes involved in transport across the blood brain barrier (BBB) highlighting potential dysfunction. Furthermore, we found a significant upregulation of integrin β4 (ITGB4) (fc = 1.1,5 padj = 9.07 × 10^11^), a vital ECM protein for BBB formation (Su et al. 2008). Interestingly, ITGB4 is among the 10 genes significantly correlated with age in the human prefrontal cortex and has also been linked to astrocyte reactivity and inflammation in rodents (Fasen et al. 2003; Milner and Campbell 2006). Neuroinflammation is associated with the aging in the CNS and many of the upregulated pathways were associated with cytokine and chemokine signalling, with a significant upregulation of CXCL1, CXCL8, CXCL6, CD70, IL-11, LIF and SERPING1, among others (Supp Fig. 4a).

To investigate whether age-related gene expression changes were accompanied by phenotypic changes, we examined if astrocytes with short telomeres displayed hallmarks of aging. Prior studies have reported that astrocytes undergo cell-type specific changes associated with aging, such as an increase in cell size and higher GFAP expression (Gatto et al. 2021; Sofroniew and Vinters 2010). In our study, we found that astrocytes with short telomeres displayed both an enlarged cell size and elevated GFAP expression (Fig. 4f, g). These observations, combined with the increased expression of cytokines, along with upregulated A1-reactive markers, complement 3 (C3) (fc = 2.93, padj = 0.021), and SERPING1 (fc = 1.02, padj = 5.85 × 10^–14^), further highlights the aged characteristics of these astrocytes and provides further support to the hypothesis that aged astrocytes develop a partially reactive phenotype (Clarke et al. 2018; Allen et al. 2023).

As DNA damage response genes were observed to be upregulated in TERT low astrocytes, we examined DNA double-strand breaks in these cells. By quantifying the number of phosphorylated H2AX (γH2AX) foci we found that TERT low astrocytes had a higher number of γH2AX foci, indicative of increased DNA damage (Fig. 4h). While the RNA seq analysis did not reveal dysregulation in cellular senescence pathways, it is worth noting that astrocytes have been demonstrated to undergo cellular senescence in vitro and in age-associated neurodegenerative diseases (Cohen and Torres 2019). In light of this, we conducted immunocytochemistry to examine p21 expression, a well-known marker of cellular senescence. Surprisingly, TERT low astrocytes exhibited an increase in p21 expression (Fig. 4i), suggesting the presence of senescence-related changes despite the absence of significant pathway dysregulation in the RNA seq data. Together, these findings provide strong evidence that telomere shortening accelerates aging processes and induces age-related changes in astrocytes.

## Discussion

Aging is the main risk factor for many neurodegenerative diseases, so understanding and recapitulating age-related characteristics in cells of the CNS is of utmost importance. There is a lack of suitable human in vitro models that mimic the functional features of neural cell aging. Here we demonstrate a successful strategy to induce aging features in human induced pluripotent stem cell (hiPSC)-derived motor neurons and astrocytes through reducing telomerase activity and telomere shortening. These hiPSCs possess telomeres measuring 5–6 kb in length. Notably, a telomere length of 5 kb has previously correlated with individuals aged 60 and above, and has been identified as a critical threshold linked to a heightened risk of mortality in a study examining leukocyte telomere length across the human lifespan (Steenstrup et al. 2017). We show that these manipulated cells exhibit key age-related phenotypes, including increased DNA damage, cellular senescence, inflammation, synaptic dysregulation and age-associated cellular morphology changes. We observe cell type-specific changes, highlighting the importance of considering cell-specific aging signatures.

It is widely accepted that the reprogramming of somatic cells into hiPSCs resets the cellular age resulting in an embryonic-like state which persists in downstream differentiated neural cells (Lapasset et al. 2011; Huh et al. 2016). There have been multiple strategies to artificially induce aging in hiPSC based models, for example Miller et al., showed progerin expression in hiPSC-derived neurons induced aging associated characteristics and enhanced phenotypes of Parkison’s disease (Miller et al. 2013). However, the question of whether the aging phenotype caused by progerin resembles physiological or pathological aging remains unclear. Telomere shortening is more closely associated with physiological aging, and a previous study generated hiPSC-derived neurons with shorter telomeres using telomerase inhibitor BIBR1532 (Vera et al. 2016). These neurons did indeed show age-like characteristics including increased DNA damage, mitochondrial ROS and dendrite atrophy. However, there are limitations to using small molecules to regulate telomerase activity, and another study found BIBR1532 was unable to induce telomerase inhibition in hiPSC-derived motor neurons (Pandya et al. 2021). To our knowledge, this study presents the first genetically modified hiPSC lines with reduced telomerase activity and shortened telomeres and thus provides a unique genetic model to induce aging characteristics.

An alternative approach to bypass the limitation of the embryonic-like state of hiPSC is the direct reprogramming of somatic cells into the mature cell type of interest, retaining cellular age. Various research groups have developed induced neuronal cells (iN) and multipotent induced neural precursor cells (iNPCs) by different methods (Mertens et al. 2015; Meyer et al. 2014; Yoo et al. 2011; Pfisterer et al. 2011; Li et al. 2015; Ring et al. 2012). Fibroblast-derived iNs preserve features of human aging including transcriptomic, epigenetic, mitochondrial and nuclear pore changes (Herdy et al. 2019; Huh et al. 2016; Kim et al. 2018; Mertens et al. 2015). Comparable transcriptional signatures were also observed in neurons with shortened telomeres, including dysregulation of genes involved in DNA damage, cellular senescence, synaptic function and synaptic plasticity. Human derived astrocytes have also been generated from tripotent iNPCs directly converted from adult fibroblast (Meyer et al. 2014). These astrocytes (iNPC-A) retain age-associated features such as accumulation of DNA damage, altered nuclear compartmentalisation and oxidative stress (Gatto et al. 2021). There is a significant overlap of cellular changes with iNPC-A and the astrocytes with shortened telomeres in the study, including increased GFAP expression, increased cell size, increased DNA damage and a heightened inflammatory response. These direct reprogramming studies have also highlighted an age-related dysregulation of nucleocytoplasmic transporters, with a decrease in karyopherin RANBP17 shown in both neuron and astrocytes (Mertens et al. 2015; Gatto et al. 2021). However, this was not observed in our aging induced model, which could be accountable to subtype differences in neuronal and astrocyte populations.

Astrocytes, the largest group of glial cells in the CNS, play a crucial role in maintaining essential neural functions. As recent studies have suggested glial cells, including astrocytes, may be more susceptible to the effects of aging compared to neurons (Zhang et al. 2021; Allen et al. 2023), we were interested to see the effect of shortened telomeres in this cell-type. Aging has been found to induce significant transcriptional changes (Soreq et al. 2017; Pan et al. 2020; Boisvert et al. 2018) and functional alterations in astrocytes (Clarke et al. 2018; Verkerke et al. 2021), impairing their ability to regulate CNS homeostasis and to provide support to neurons. It has been reported that astrocytes become more reactive with age and this is accompanied with an increase in GFAP expression that has shown to correlate with age (Boisvert et al. 2018; Clarke et al. 2018; Nichols et al. 1993; Porchet et al. 2003; Wruck and Adjaye 2020). GFAP was the most upregulated protein in a proteomic analysis of 2 and 24 month-old rat spinal cords (Lee et al. 2017). Indeed, astrocytes with shortened telomeres displayed an increase in GFAP expression and significant hypertrophy, correlating with age-related and reactive morphological changes (Sofroniew and Vinters 2010; Jyothi et al. 2015; Escartin et al. 2021). Furthermore, another feature of aged astrocytes is an increase in production of cytokines, and astrocytes with shortened telomeres show an upregulation of cytokines and chemokines supporting the theory of increased neuroinflammation with aging. Interestingly, a study that compared cytokine release in young and old spinal cords, found only 3 cytokines to be increased in old spinal cords, with an increase in cytokine sICAM-1 (Piekarz et al. 2020), which was found to have increased expression in astrocytes with shortened telomeres in this study, which have a spinal cord lineage.

Different cell types have been shown to display cell-type specific aging signatures (Zhang et al. 2021). Upon comparing common DEGs between motor neurons and astrocytes with shortened telomeres, the majority of the DEGs were found to be cell type specific, with 91.4% of DEGs motor neuron specific and 81.1% astrocyte specific. However, common dysregulated genes and pathways were observed, which could be used to gain insights into cross-cellular interactions involved in spinal cord aging. Common dysregulated pathways include synaptic function, axon development and guidance, and extracellular matrix component and organisation. Notably, common dysregulated ECM genes including collagen A2 (COL1A2), elastin (ELN), Vitronectin (VTN), glycoprotein tenascin-R (TNR) and Vascular Cell Adhesion Molecule 1 (VCAM1). A study examining molecular changes in spinal cord aging in mice also identified significant alterations in ECM components, such as collagens and matrix metalloproteinases (MMPs), which were accompanied by changes in spinal cord structure (Piekarz et al. 2020). Together, this supports an essential role of ECM changes in spinal cord aging and its significance should be further investigated.

Over the last 10 years, there has been increasing evidence for non-telomeric, non canonical functions of TERT (Thompson and Wong 2020). For example, it has been shown TERT can undergo nucleocytoplasmic shuttling and localise to the mitochondria altering respiration, although the exact mechanisms are unknown (Haendeler et al. 2009). There is conflicting evidence about the expression and localisation of TERT in neurons, which could be accounted for by different neuronal subtypes (Eitan et al. 2016). However, it is widely accepted that TERT is highly expressed at embryonic stages in neural progenitor cells and the expression gradually declines during development (Martín-Rivera et al. 1998; Ishaq et al. 2016), which we observed in this study. Several studies have shown that TERT is also expressed in adult neural stem cell (NSCs) populations in the brain (Lee et al. 2012; Limke et al. 2003). In both embryonic and adult NSC populations, TERT has shown to have an essential role in both proliferation and neuronal differentiation, although there is conflicting evidence if TERT activity encourages or inhibits differentiation into a neuronal lineage (Richardson et al. 2007; Schwob et al. 2008; Ferrón et al. 2009; Mattson et al. 2001). In this study, while we emphasise the crucial involvement of TERT and short telomeres in proliferation and neuronal differentiation in NPCs, the interplay between these factors makes it challenging to distinguish their individual contributions. Further investigation is needed to find out which specific outcomes can be attributed to TERT, short telomeres, or their combined influence. The strategy of inducing aging features in hiPSC-derived neural cells through decreasing telomerase activity and telomere shortening offers valuable insights into age-related phenotypes. This model provides a renewable source of genetically modified cells that can be differentiated into many different cell types, enabling telomere shortening and age-associated changes to be studied in a cell-type specific manner. Furthermore, this approach holds promise for investigating the relationship between aging and age-related diseases by reducing telomerase activity in hiPSC from a disease background. However, it is essential to acknowledge the limitations of this model. Telomere shortening represents just one aspect of cellular aging, and while it sheds light on specific aging pathways, it may not fully recapitulate the complexity of the aging process in the CNS. Additionally, the accelerated aging phenotype resulting from reduced telomerase activity may not fully represent the progressive changes that occur during natural aging. Nevertheless, despite these limitations, the study offers valuable insights into the impact of telomere shortening in neurons and astrocytes and provides a useful platform to investigate aging features in the CNS and age-associated neurodegenerative diseases.

## Methods

### hiPSC cell culture

hiPSC lines were cultured feeder-free on Matrigel-coated dishes with StemMACS™ iPS-Brew XF (Miltenyi Biotec) according to product’s instructions. Routine passaging using ReLeSR (Stem Cell Technologies) was performed once every 4 days. The parent hiPSC line used was BJ-hiPSC (Ng et al. 2015).

### Generation of hiPSC-derived motor neurons

hiPSCs were differentiated into spinal motor neuron progenitor cells and spinal motor neurons using a previously published protocol (Hor et al. 2021). The hiPSCs were neuralized by treating them with CHIR99021 (4.25 μM) and LDN-193189 (0.5 μM) day 0–10. Retinoic acid (1 μM) was added from day 3 to 16 to induce caudalization, and purmorphamine (2 μM), a sonic hedgehog (SHH) activator, was added from day 11 to 16 to promote ventralization. From day 17 onwards (D17–D28), the cultures were supplemented with brain-derived neurotrophic factor (BDNF) (10 ng/ml), glial cell-derived neurotrophic factor (GDNF) (10 ng/ml), and ascorbic acid (200 μM) to facilitate neuronal maturation. The cells were cultured throughout the differentiation process in N2B27 media, which consisted of a mixture of MACS Neuro Medium (50%), DMEM/F12 medium (50%), N2, MACS NeuroBrew-21, 1 × Non-Essential Amino Acids (NEAA), and 1 × GlutaMAX.

### Generation of hiPSC-derived astrocytes

To generate hiPSC-derived astrocytes, 1 × 10^6^ hiPSCs were plated onto a 10 cm dish in StemMACS™ iPS-Brew XF (Miltenyi Biotec) and ROCK inhibitor Y-27632. The following day, the hiPSC were treated with neural induction media for 3 days (D0–D3); N2B27 supplemented with 0.5 μM LDN193189 and 4.25 μM CHIR99021. On day 3, the neural progenitors were plated at 200,000 cells per well onto matrigel-coated 6-well plates. Media was changed to N2B27 supplemented with 10% FBS, 0.5 μM LDN193189, 4.25 μM CHIR99021 and 1 μM retinoic acid (D3–D10). On day 10 (or before if cells are confluent) glial progenitors were split (1:2) with Accutase. On day 10, cells were treated with N2B27 supplemented with 10% FBS, 1 μM of retinoic acid, 1 μM purmorphamine (D10–D18). On day 18, glial progenitors are treated with media N2B27 supplemented with 10% FBS, 10 ng/ml GDNF and 10 ng/ml BDNF. Media was changed every other day. Astrcoytes were analysed on D35 and D40. The cells were cultured throughout the differentiation process in N2B27 media; MACS Neuro Medium (50%), DMEM/F12 medium (50%), N2, MACS NeuroBrew-21, 1 × Non-Essential Amino Acids (NEAA), and 1 × GlutaMAX.

### Generation of hiPSC-derived spinal organoids

hiPSCs were differentiated into spinal cord organoids using a previously published protocol (Hor et al. 2018). Briefly, the differentiation process followed the same small molecule treatment as 2D motor neuron differentiation, with a few modifications. On day 0 of differentiation, cells were seeded in a 96-well round bottom low attachment plate format, with 2 × 10^5^ cells per well. On day 10, the cells were encapsulated with Matrigel (Corning). From day 14 onwards, the cultures were transferred to spinner flasks (Corning) and maintained up to day 42. Cryosectioning of the organoids was performed on days 28, 35, and 42.

### Plasmids and cloning

Guide RNAs (gRNA) were designed using the gRNA design web tools by Feng Zhang’s lab: crispr.mit.edu and the benchling software, https://www.benchling.com. The gRNA CCACGTTCGTGCGGCGCCTG targeting exon one of TERT (ENST00000310581.10) was cloned into a CRISPR/ Cas9 GFP expression vector pSpCas9(BB)-2A-GFP (PX458) (Addgene plasmid #48138).

### Transfection of hiPSC line

hiPSC were seeded onto a 6 well plate at a density of 2 × 10^5^ cells. gDNA (1.25 µg) was incubated with Lipofectamine Stem Transfection Reagent (8 μl) (Thermo Fisher) and Opti-MEM™ (250 μl) (Thermo Fisher) according to the manufacturer’s protocol. The solution was then added to the hiPSCs in StemMACS™ iPS-Brew XF (2 ml) (Miltenyi Biotec) dropwise. The live cells were harvested 1–3 days later and GFP + single cells via flow cytometry onto Matrigel coated plates containing StemMACS™ iPS-Brew XF (Miltenyi Biotec) and ROCK inhibitor Y-27632 (Stem Cell Technologies). Individual cells grew into colonies before being picked and passaged into an individual well per colony. The clones are validated via sequencing of the genomic DNA of the colony.

### Genomic DNA extraction and sequencing

Genomic DNA (gDNA) was extracted from samples using the NucleoSpin Tissue, Mini kit (Macherey–Nagel) according to the manufacturer’s protocol. The NanoDrop 1000 spectrophotometer (Thermo Fisher Scientific) was used to quantify DNA concentration.

DNA sequencing was performed using 200–500 ng of DNA as a template in a 25 μl PCR reaction with GoTaq Green Master Mix (Promega) with forward primer CGTCCTCCCCTTCACGTC and reverse primer CTCCTTCAGGCAGGACACCT. The PCR products were purified using the QIAquick PCR Purification Kit (Qiagen). For cycle sequencing, 3–5 ng of DNA was combined with BigDye Terminator v3.1 (Thermo Fisher), forward or reverse primers (10 μM), and nuclease-free water. The reaction underwent thermocycling with initial denaturation at 96 °C for 1 min, followed by 30 cycles of denaturation at 96 °C for 10 s, annealing at 50 °C for 5 s, and extension at 60 °C for 4 min. Sequencing analysis was performed using FinchTV 1.4 and CLC Genomics Workbench software. The samples were sent to the DNA Sequencing Facility at IMCB, A*Star, Singapore.

### RNA extraction and sequencing

RNA from Day 10 NPCs (WT, TERT C1, TERT C2, n = 3), Day 28 motor neurons (WT, TERT C2, n = 3) and D35 astrocytes (WT, TERT C1, TERT C2, n = 2) were extracted using the RNeasy Mini Kit (Qiagen, Germany, #74104) with RNA clean-up. Day 28 motor neurons were enriched for neurons by magnetic cell sorting using MACS cell separation antibodies, microbeads, and reagents (Miltenyi Biotec) targeting CD171 ± PSA±NCAM + cells. For the Day 10 NPCs, WT, C Three biological replicates per sample were used, and the total RNA samples were sent to Axil Scientific Pte. Ltd. (Singapore) for RNA sequencing (RNA-seq) on the Illumina HiSeq platform, following the standard paired-end protocol. Astrocyte samples were sent to Novogene (Singapore).

RNA-seq data quality was monitored via the FASTQC package (https://www.bioinformatics.babraham.ac.uk/projects/fastqc/). Reads pre-processing was performed by trim_galore (version 0.6.5-1, https://github.com/FelixKrueger/TrimGalore). Mapping of RNA-seq reads was done using STAR_2.5.4a (Dobin et al. 2013) with default parameters for RNA-seq data; RSEM software (Li and Dewey 2011) were used to quantify the gene-level expression. Deseq2 (http://www.bioconductor.org/packages/release/bioc/html/DESeq2.html) package was utilised for differential gene expression analysis. Pathway enrichment analysis was performed using the Metascape (Zhou et al. 2019).

### Absolute telomere length qPCR assay

The average telomere length was determined using the Absolute Human Telomere Length Quantification qPCR Assay Kit (ScienCell, #8918). This kit utilises telomere and single reference copy (SRC) primers to amplify telomere sequences and a reference genomic DNA with a known telomere length. For the qPCR, 5 ng of genomic DNA was combined with the telomere or SRC primers, 2 μl from the stock solution, 10 μl of 2 × , GoldNStart TaqGreen qPCR master mix (provided by the kit), and nuclease-free water to a final volume of 20 μl. Each sample was prepared in triplicates in a 384-well format and cycled on the QuantStudio 5 Real-Time PCR System (Applied Biosystems). The cycling steps included an initial denaturation at 95 °C for 10 min, followed by 32 cycles of denaturation at 95 °C for 20 s, annealing at 52 °C for 20 s, and extension at 72 °C for 45 s. Melt-curve analysis was performed at the end of the run. To calculate the average telomere length, the ΔCq (TEL) and ΔCq (SCR) values were determined by subtracting the Cq values of the target sample from the Cq values of the reference sample for the telomere and SRC primers, respectively. Then, ΔΔCq was calculated as ΔCq (TEL)–ΔCq (SCR). The total telomere length per diploid cell was calculated using the formula: Reference sample telomere length (1.23 ± 0.09 Mb) × 2^(−ΔΔCq)^. Since there are 92 chromosome ends in one diploid cell, the average telomere length on each chromosome end was obtained by dividing the total telomere length by 92.

### Telomerase repeat amplification protocol (TRAP) assay

The TRAP assay, based on the protocol by Mender and Shaay (Mender and Shay 2015), was employed to visualise telomerase activity in the cells. This assay involves the extension of a telomere substrate by endogenous telomerase. The extended products are then amplified using the TS upstream primer and the ACX downstream primer, along with a 36-bp internal standard control (TSNT), in a PCR reaction. Samples were prepared in 1 × , 10 × , and 100 × dilutions. Negative controls consisted of the PCR solution with a lysis buffer. The resulting products were separated on a 10% non-denaturing acrylamide gel at 210 V for 40 min and visualised by staining the gel with SYBR Gold Nucleic Acid Gel Stain (Thermo Fisher) for 10 min. Gel images were captured using the ChemiDoc XRS + System (Bio-Rad Laboratories) and analysed with Image Lab Software (Bio-Rad Laboratories). Blots were quantified using the following formula [(TRAP sample-negative control)/Internal control of sample]/[(WT sample (positive control)-negative control)/Internal control of WT sample)]*100.

### Telomere FISH analysis

The cells on the slide were fixed with 4% paraformaldehyde (PFA) at RT for 10 min. The slide was washed with 1 × PBS three times. The hybridization buffer was prepared with 20 mM Tris (pH 7.4), 60% Formamide, 5% Blocking Reagent (Roche 11096176001) and 500 nM Telomeric DNA Probe (PNABio. F1002 TelC-Cy3). The hybridization buffer was added to the slide and the slide was heated for 10 min at 85 °C. The slide was placed at RT in the dark for hybridization. After 2 h of hybridization, the slide was washed with wash solution (2 × , saline-sodium citrate (SSC) buffer + 0.1% Tween-20) for 10 min twice at 55 °C and once at room temperature. The DAPI solution was added to the slide. After 10 min, the slide was washed with 2 × SSC, 1 ×SSC, and finally with water for 2 min each. Finally, the slide was mounted with mounting media (Prolong Gold antifade, Thermo Fisher Scientific, Cat#P36930) and observed in fluorescence microscopes with appropriate filters.

### SDS-PAGE and Western blot (WB)

Protein lysates were resolved on 10–12% SDS-PAGE gels and transferred to a nitrocellulose membrane using the Trans-Blot Turbo Transfer System (Bio-Rad). The membranes were then blocked in 5% milk in TBST buffer (Tris-buffered saline with 0.1% Tween 20) and incubated overnight with primary antibodies (Supp Table 1) diluted in 5% milk in TBST buffer. After incubation, the membranes were washed three times with TBST buffer and probed with horseradish peroxidase secondary antibodies (Life Technologies) diluted in 5% milk in TBST buffer. Subsequently, the membranes were washed three times with TBST buffer before being treated with the ECL Western Blotting Substrate (Bio-Rad). The visualisation of protein bands was performed, and band intensities were analysed using the Image Lab Software (Bio-Rad Laboratories).

### Immunocytochemistry, image acquisition and image analysis

Cells were fixed with 4% PFA for 15 min at room temperature. Then blocked and permeabilized in 5% BSA and 0.1% Triton X-100 in PBS for 1 h. Primary antibodies (Supp Table 1) were diluted in 5% BSA in PBS and incubated overnight at 4 °C. The cells were washed 2 times in PBS and incubated in secondary antibodies diluted 1:1000 in 5% BSA in PBS for 1 h. Cells were incubated with DAPI for 10 min and washed 2 times with PBS. Cells were visualised using the Opera Phenix^®^ Plus High Content Screening System (Perkin Elmer) using the 20 × and 40 × water objective. Image analysis was performed in the Columbus Image Data Storage and Analysis System (Perkin Elmer).

Cryo-sectioned slides were air-dried at 37 °C for 5 min, permeabilized with 0.5% Triton X-100 in PBS for 30 min, and blocked with 5% FBS in PBS for 1 h. Primary antibodies were diluted in the blocking buffer and incubated overnight at 4 °C. After washing with PBS, sections were incubated with a 1:1500 dilution of secondary antibody in the blocking buffer for 1 h at room temperature. DAPI stain was added for 10 min to visualise nuclei. Slides were washed, mounted on coverslips using VECTASHIELD Mounting Medium, and sealed with clear nail polish. Visualisation and analysis were performed using the Nikon Ti-E; Zyla microscope.

#### Annexin V apoptosis assay

The apoptosis assessment was conducted following the guidelines provided by the manufacturer’s protocol, utilizing the PE Annexin V Apoptosis Detection Kit I from BD Biosciences. Approximately 1 × 10^6^ cells were suspended in 1 ×  Binding Buffer. For each sample in the assay, a solution containing 1 × 10^5^ cells (100 μl) was employed. Subsequently, 5 μl of PE Annexin V and 5 μl of 7-AAD from the kit were introduced into the solution. The cells were left to incubate at room temperature in the dark for 15 min. Following the incubation, 400 μl of 1 ×  Binding Buffer was added to each tube, and the cells were then analyzed using flow cytometry. The cells were classified as follows: viable cells (negative for both PE Annexin V and 7-AAD) and dead cells (positive for 7-AAD and negative for PE Annexin V).

### Statistical analyses

All data is represented as at least three biological replicates, unless stated otherwise. Statistical significance between samples were determined using the appropriate statistical test. P value is shown as; *P* > 0.05 = ns, *P* < 0.05 = *, *P* < 0.01 = **, *P* < 0.001 = ***, *P* < 0.0001 = ****.

### Supplementary Information

Below is the link to the electronic supplementary material.

Supplementary file1 (DOCX 1044 KB)

## Data Availability

Raw and processed dataset from RNA-Seq of NPCs, motoneurons and astrocytes are available on the Gene Expression Omnibus database: GSE240447.
