# Supplementary material for: Telomere shortening induces aging-associated phenotypes in hiPSC-derived neurons and astrocytes
Source: Biogerontology. 2023 Nov 21;25(2):341–60. doi: 10.1007/s10522-023-10076-5 (PMC10998800; doi:10.1007/s10522-023-10076-5)
Supplement: Supplementary file 1 — Supplementary file1 (DOCX 1044 KB) [file 10522_2023_10076_MOESM1_ESM.docx]

**Supplementary Figures**

**Supplementary Figure 1**

**
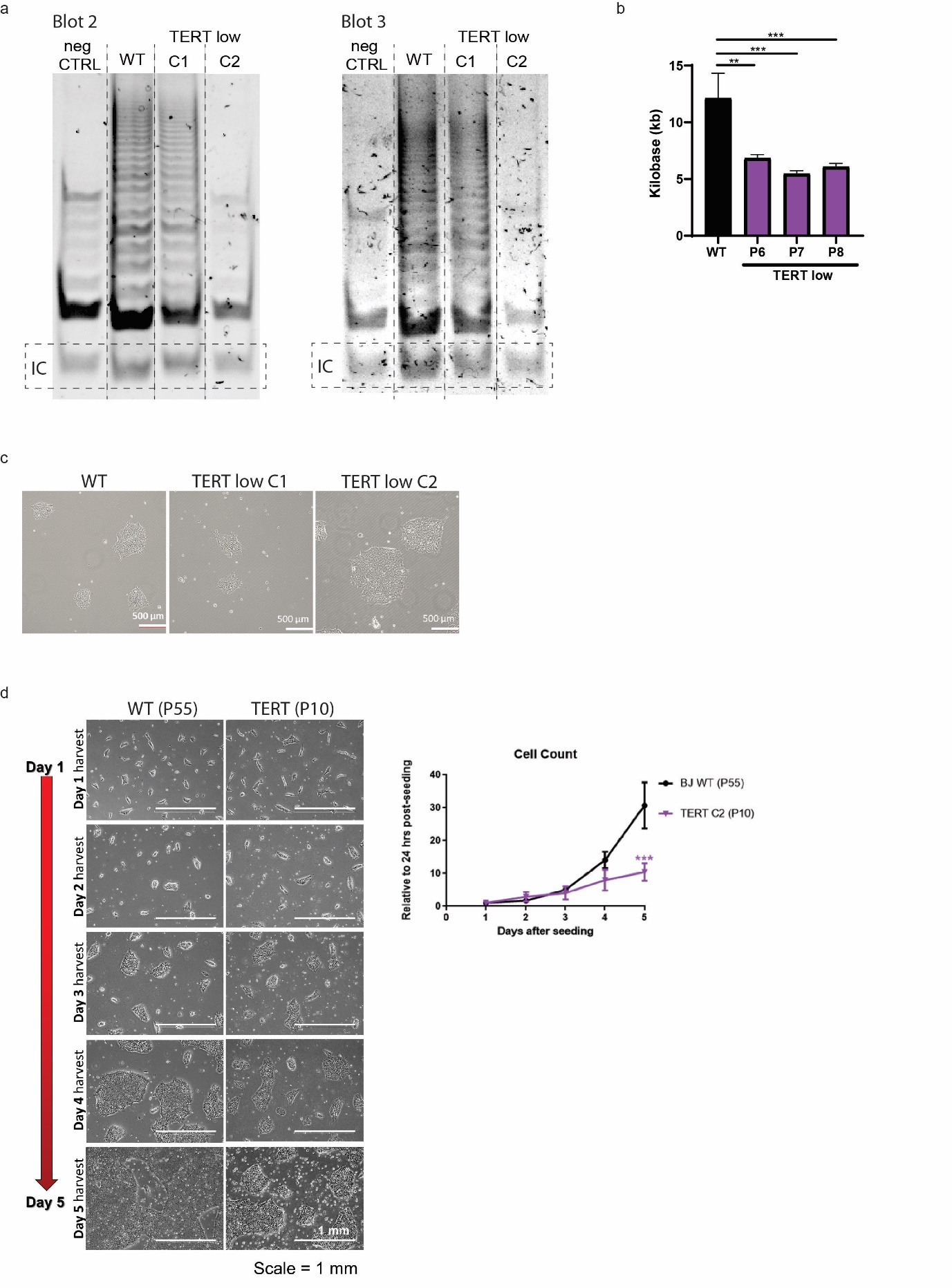
**

**Supplementary Figure 1**

(a) Additional TRAP assay blots that were used for quantification of TRAP activity in Figure 1c.

(b) Average telomere length of WT and TERT low C2 hiPSC over 3 cell passages (P6, P7 and P8) using the Absolute Human Telomere Length Quantification qPCR Assay Kit. Shows shorter telomeres in TERT low C2 are maintained over subsequent passages. Data is shown as mean ± SD, n=3, **P < 0.01 and ***P < 0.001; ANOVA with Tukey’s multiple comparisons test.

(c) Brightfield images of hiPSC clones. Scale bar = 500μm.

(d) Brightfield images of WT and TERT low C2 hiPSC over 5 days. Quantification of the number of cells each day in culture normalised to 24 hrs post seeding. TERT low C2 shows a significant reduction in proliferation. Data is shown as mean ± SD, ***P < 0.001; 2way ANOVA with Sidak’s multiple comparisons test.

**Supplementary Figure 2**


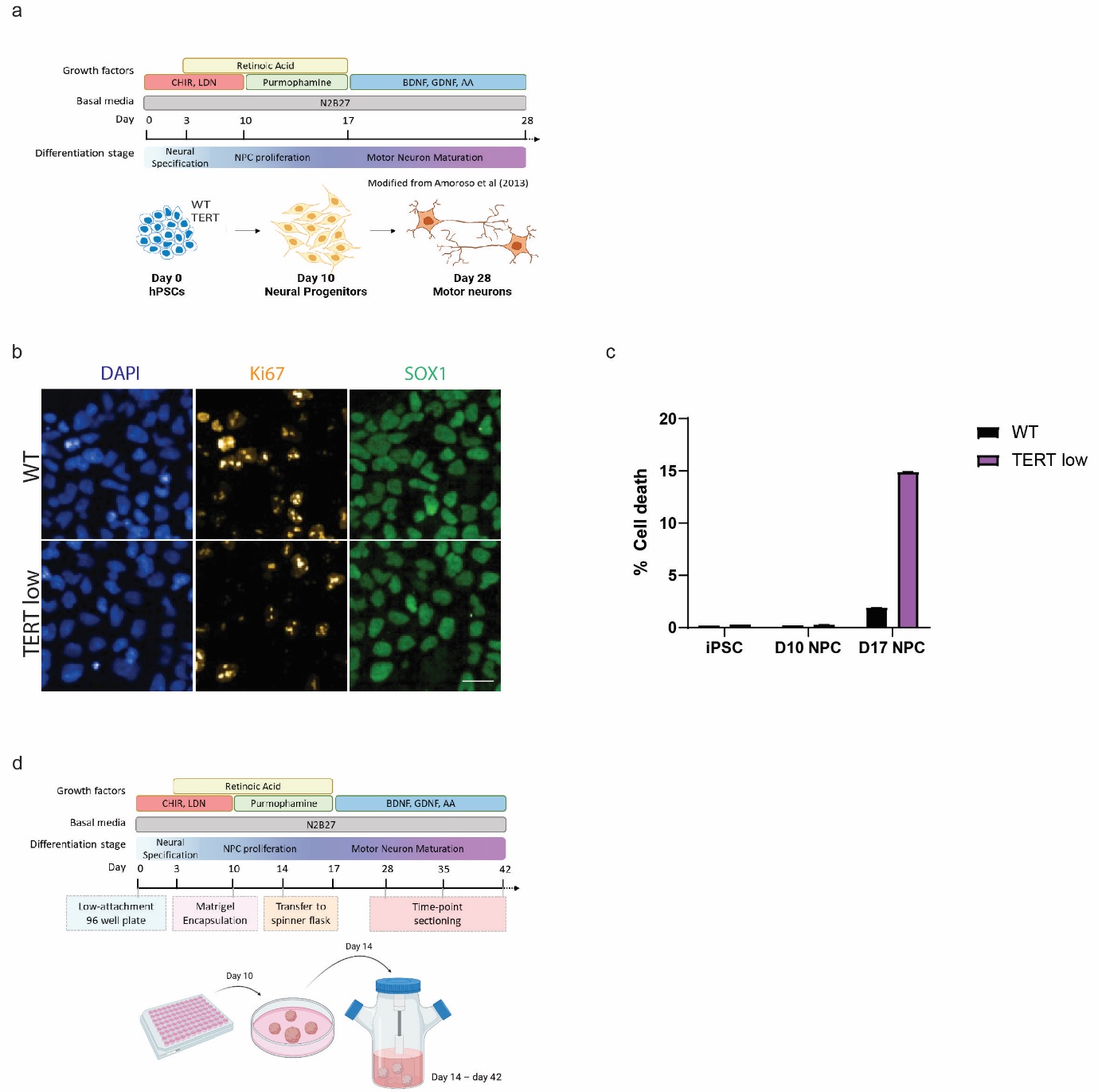


**Supplementary Figure 2**

(a) Neuron progenitor and motor neuron differentiation protocol. Schematic of motor neuron differentiation from iPSCs. Neural progenitor cells (NPCs) are obtained on day 10 whilst motor neurons are obtained on day 28 of differentiation.

(b) Representative immunostained images of Ki67, SOX1 and DAPI in NPCs show reduced proliferation in TERT low NPCs.

(c) Quantification of cell death using an Annexin V apoptosis assay at different timepoints of motor neuron differentiation show TERT low NPCs have an increased percentage of dead cells (PE Annexin V-, 7-AAD+) at day 17. Data is shown as mean ± SD, n=3.

(d) Schematic of spinal organoid generation.

**Supplementary Figure 3**


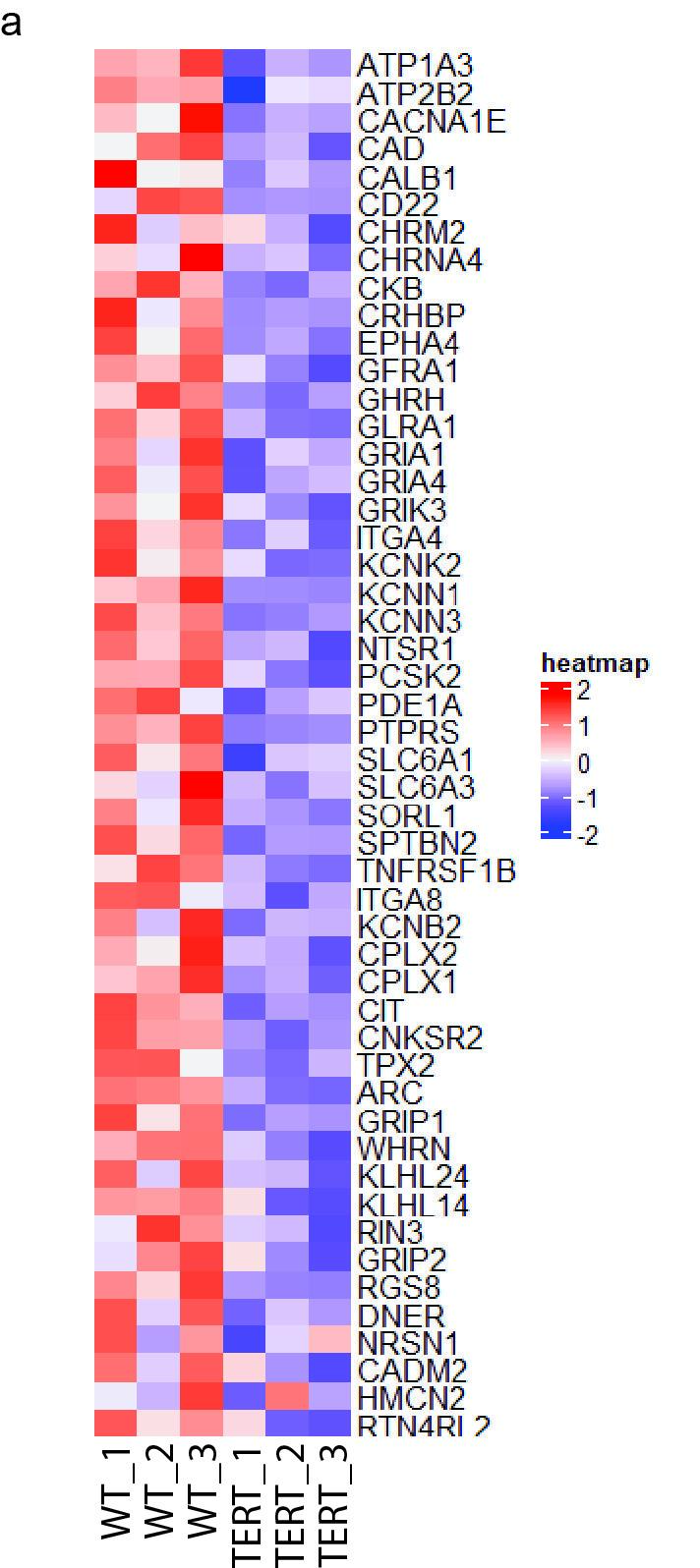


**Supplementary Figure 3**

(a) Down regulated genes in TERT low motor neurons related to the neuronal cell body.

**Supplementary Figure 4**


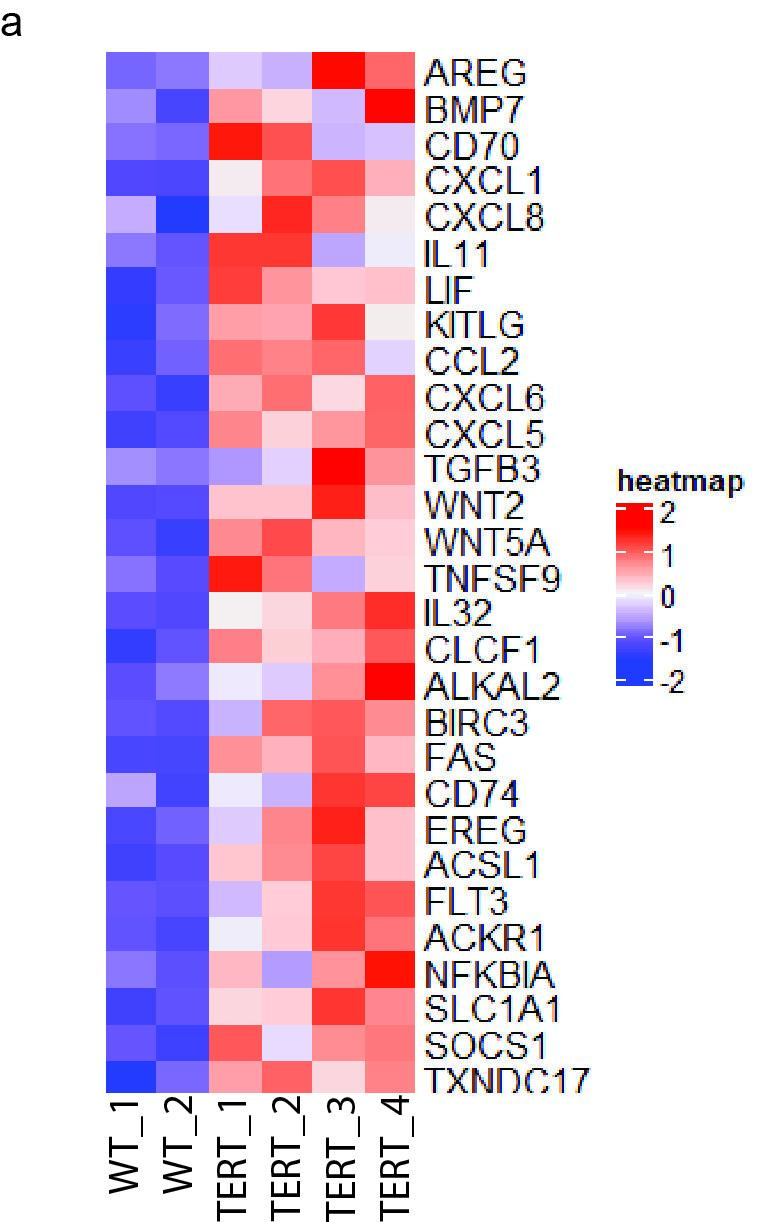


**Supplementary Figure 4**

(a) Up regulated inflammation related genes in TERT low astrocytes.

**Supplementary Table 1. Antibodies used in the study**

| **Antibody** | **Company** | **Catalog No.** | **Dilution** | **Use** |
| --- | --- | --- | --- | --- |
| NANOG | CST | 3580 | 1:1000 | ICC |
| OCT4 | SCBT | sc-5279 | 1:500 | ICC |
| SOX1 | R&D systems | AF3369 | 1:500 | ICC |
| HOXB4 | Abcam | ab133521 | 1:200 | ICC |
| cCASP3 | CST | 96616 | 1:1000 | ICC |
| SMI32 | BioLegend | 801701 | 1:1500 | ICC |
| KI67 | Abcam | ab16667 | 1:1000 | ICC |
| ISL1 | Abcam | ab109517 | 1:500 | ICC |
| P21 | CST | 2947 | 1:500 | ICC |
| CD44 | CST | 3570 | 1:500 | ICC |
| GFAP | Abcam | ab7260 | 1:500 | ICC |
| γH2AX | Abcam | ab11174 | 1:2000 | ICC |
| TUJ-1 | BioLegend | 801202 | 1:1000 | ICC |
| γH2AX | Abcam | ab11174 | 1:500 | WB |
| β-Actin | SCBT | sc-47778 | 1:1000 | WB |
